# Dosimetric effect due to the motion during deep inspiration breath hold for left‐sided breast cancer radiotherapy

**DOI:** 10.1120/jacmp.v16i4.5358

**Published:** 2015-07-08

**Authors:** Xiaoli Tang, Tim Cullip, John Dooley, Timothy Zagar, Ellen Jones, Sha Chang, Xiaofeng Zhu, Jun Lian, Lawrence Marks

**Affiliations:** ^1^ Medical Physics Department Memorial Sloan Kettering Cancer Center West Harrison NY; ^2^ Department of Radiation Oncology University of North Carolina Chapel Hill Chapel Hill NC USA

**Keywords:** DIBH, motion management, delivered dose, left‐sided breast cancer, heart dose sparing, IMN

## Abstract

Deep inspiration breath‐hold (DIBH) radiotherapy for left‐sided breast cancer can reduce cardiac exposure and internal motion. We modified our in‐house treatment planning system (TPS) to retrospectively analyze breath‐hold motion log files to calculate the dosimetric effect of the motion during breath hold. Thirty left‐sided supine DIBH breast patients treated using AlignRT were studied. Breath‐hold motion was recorded — three translational and three rotational displacements of the treatment surface — the Real Time Deltas (RTD). The corresponding delivered dose was estimated using the beam‐on portions of the RTDs. Each motion was used to calculate dose, and the final estimated dose was the equally weighted average of the multiple resultant doses. Ten of thirty patients had internal mammary nodes (IMN) purposefully included in the tangential fields, and we evaluated the percentage of IMN covered by 40 Gy. The planned and delivered heart mean dose, lungs V20 (volume of the lungs receiving >20 Gy), percentage of IMN covered by 40 Gy, and IMN mean dose were compared. The averaged mean and standard deviation of the beam‐on portions of the absolute RTDs were 0.81±1.29 mm, 0.68±0.85 mm, 0.76±0.85 mm, 0.96°±0.49°,0.93°±0.43°, and 1.03°±0.50°, for vertical, longitudinal, lateral, yaw, roll, and pitch, respectively. The averaged planned and delivered mean heart dose were 99 and 101 cGy. Lungs V20 were 6.59% and 6.74%. IMN 40 Gy coverage was 83% and 77%, and mean IMN dose was 4642 and 4518 cGy. The averaged mean motion during DIBH was smaller than 1 mm and 1°, which reflects the relative reproducibility of the patient breath hold. On average, the mean heart dose and lungs V20 were reasonably close to what have been planned. IMN 40 Gy coverage might be modestly reduced for certain cases.

PACS number: 87.55.km, 87.55.N

## I. INTRODUCTION

The anticancer effects of radiation for patients with left‐sided breast cancer might be partly negated by radiation therapy (RT)‐associated cardiac toxicity.^1^, ^2^, ^3^ Since the heart can be displaced medially, inferiorly, and posterior (i.e., away from the left breast) during deep inspiration in most patients, one approach to reduce incidental cardiac irradiation is to treat patients specifically during this portion of the respiratory cycle (e.g., using DIBH). This approach allows one to typically maintain coverage of the target tissues yet markedly reduce the degree of incidental cardiac irradiation.^4^, ^5^, ^6^, ^7^, ^8^, ^9^, ^10^, ^11^, ^12^, ^13^, ^14^

AlignRT (Vision RT Ltd., London, UK) has been used in our clinic for routine DIBH implementation. It continually tracks the position of multiple points on the patient's surface within the treated area and thus assesses variations in both translational and rotational displacements during treatment. We herein use the information stored from our patients treated with AlignRT to quantify the degree of breath‐hold motion and its dosimetric consequences.

Although there were reports on the breath‐hold motion using various systems,^15^, ^16^ based on our knowledge, no one has reported the corresponding dosimetric effect. We modified our in‐house treatment planning system (TPS) so that it can process the actual patient motion information and calculate the dosimetric variation due to the motion. We describe how the calculation was done in the Materials and Methods section below. The purpose of this study was to answer a clinical question: How much dosimetric variation the breath‐hold motion might generate compared to the plan?

## II. MATERIALS AND METHODS

Details of the AlignRT beam hold system have been reported.^17^, ^18^, ^19^ Briefly, the AlignRT beam hold system consists of three ceiling‐mounted camera pods that work together to generate a 3D surface image of the patient. To set up and track patients, the system needs a reference image and a verification image. The CT skin rendering was our default reference image. Actual patient's surface was used as reference when bolus was needed for the treatment. The accuracy of the patient's surface capture was verified by port films. The system registers each verification image to the reference to determine, in real time, the differences between the reference and verification images, including three translational and three rotational displacements — the Real Time Deltas (RTD). For a given patient, the RTDs were calculated against the same reference image for the entire treatment. In other words, RTDs were absolute displacements to the same surface. Automatic gating was implemented. Tolerance levels are set (for permitted differences between the reference and verification images) for the radiation beam to be turned “on” automatically. For most patients, ±3 mm and ± 3° were used for the translational and rotational differences, respectively.

All patients were treated in the supine position. A patient is considered to have set up to the breath‐hold surface if the RTDs of the current breath‐hold region of interest (ROI) to the planned one are within predetermined tolerances. During treatment, the therapists provide audio coaching (e.g., “breathe in a tad more”) through intercom, as needed. Patient is instructed to take a deep breath and hold it, and the radiation beam is turned on automatically while the patient is holding breath and all RTDs are within the tolerance. Gated portal films are taken at least once per week, alternating between medial and lateral tangential films.

AlignRT uses an iterative closest point (ICP)‐based algorithm for surface matching. The algorithm registers the verification surface to the reference surface and calculates the shortest distance (by minimizing the mean squared error of these two surfaces). The distance is reported in the format of RTDs. We assume that the isocenter location relative to the surface does not change during the breath hold. Therefore, by knowing how much the surface deviates, we know where the isocenter has moved. The tangential beams can be moved accordingly and the delivered dose can, therefore, be calculated.

### A. Patient information

An Institutional Review Board (IRB) had been approved to retrospectively analyze DIBH patient data. The stored motion log files (that contain the translational and rotational displacement data over time) for 30 patients treated with tangential fields for left‐sided breast, delivered during DIBH, in our clinic from 2012 and 2013 were analyzed. Fractionation schedules included 200 cGy×23 fractions (11 patients), 267 cGy×16 fractions (10 patients), 200 cGy×25 fractions (8 patients), and 180 cGy×28 fractions (1 patient). The internal mammary nodes (IMN) were intended to be included in the tangential fields in 10 patients. Whenever IMN was included as part of the tangential beams, the volume of the IMN covered by 40 Gy was evaluated. If part of the IMN was not covered by 40 Gy, a separate electron boost might be needed to bring IMN to the desired dose. Electron boost is beyond the scope of this study, and will not be discussed here. Since the IMN is always at the edge of the tangential beams, even a small amount of motion might affect its coverage. Therefore, we included the IMN coverage in this study, as well.

### B. Motion (RTD) analysis

RTDs were recorded four to seven times per second, depending on the size of the ROI (longer times needed to process images for larger ROIs). A typical ROI was selected to include the external skin of the treatment area. For each patient, the RTD recording frequency is approximately the same throughout the treatment course. At each recorded instance, the three translational and three rotational RTDs were calculated, and these RTDs represented the breath‐hold motion. Since the RTDs were calculated against the same reference ROI (the default was the planning CT skin rendering) for each fraction for both setup and treatment, the interfraction variations in breath‐hold position would not propagate to treatment RTDs. The RTDs along with their corresponding beam‐on/off status were saved in a motion log file, and only the beam‐on portions were considered. For each patient, RTDs from all treatments were included, and then the mean, standard deviation, maximum, and minimum of the RTDs were calculated (for each of the three translational and three rotational parameters). The average motion (the mean and standard deviation) was calculated using the absolute values of the RTDs. The signed RTDs were used to estimate the delivered dose.

**Figure 1:**
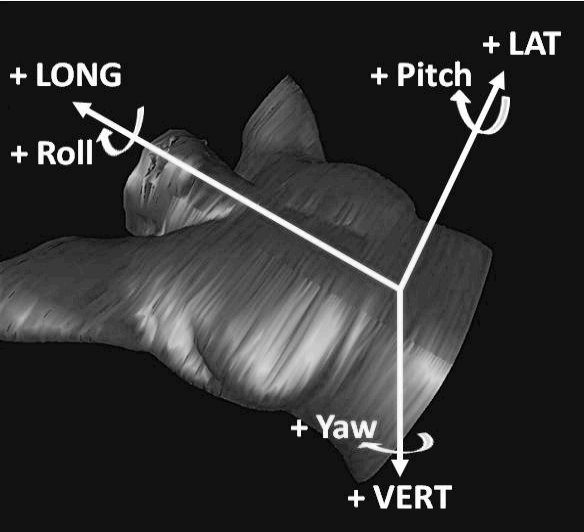
The translational and rotational orientations.

Figure 1 shows the definition of the positive vertical (VERT), longitudinal (LONG), lateral (LAT), yaw, roll, and pitch. The RTDs will be listed the same order as (VERT mm, LONG mm, LAT mm, yaw°, roll°, pitch°) throughout this work.

The average of the mean, standard deviation, maximum, minimum, absolute mean, and absolute standard deviation of the RTDs over all patients are reported, along with their standard deviations.

### C. Motion‐induced dose effect analysis

The DIBH plan was generated on the breath‐hold CT scan without considering breath‐hold motion (i.e., RTDs were zero). However, there is always a certain level of motion and thus predetermined RTD tolerances (±3 mm and ± 3°) are used clinically. Our DIBH clinical planning guidelines can be found in Tang et al.^(20)^

We modified our in‐house TPS so that the motion information from the RTD measurements can be used to recalculate a more accurate estimate of the actual delivered dose (compares to the planned dose using CT without considering any breath‐hold motion). For each position (set of recorded RTDs), the dose was recomputed using our modified in‐house treatment planning system, and the equally weighted average of the multiple resultant doses was computed. As an example, considering a total number of n positions (n sets of RTDs) recorded, the dose was recomputed for each recorded position on the original treatment plan. The final dose was the summation of all the n dose results and then divided by n. The RTDs were sampled at approximately the same rates. For each patient, the clinical patient dose statistics (delivered dose statistics) were compared to those from the treatment plan (that assumes no breath‐hold motion). Heart and lungs doses were considered for the treatment plan. We did not have clearly defined constrains for these two organs at risk (OAR). Instead, our clinicians tried to minimize the heart and lung dose while balancing the competing target coverage. Mean heart dose and lungs V20 were studied. For patients whom the IMN were being targeted, the percentage of the IMN receiving 40 Gy was considered, as this is the dose level that our clinicians usually aim to electively irradiate these nodes. More specifically, IMN 40 Gy percentage coverage and IMN mean dose were considered. Scattered plots were used to compare the planned and delivered doses. The averaged mean, standard deviation, maximum, and minimum over all patients were reported.

## III. RESULTS

### A. Motion (RTD) analysis

Table 1 shows the analysis of the RTD statistics for all 30 patients.

**Table 1:** Beam‐on portions of RTD statistics over all 30 patients

	Average±SD *(over all patients)*					
*RTDs*	*VERT (mm)*	*LONG (mm)*	*LAT (mm)*	*Yaw°*	*Roll°*	*Pitch°*
Mean	0.49±0.93	‐0.17±0.82	‐0.01±0.97	0.30±1.15	0.04±1.17	0.39±1.26
SD	0.90±0.36	0.91±0.76	0.82±0.49	0.42±0.43	0.38±0.37	0.49±0.25
Max.	2.71±0.42	2.41±0.99	2.07±0.90	1.40±1.17	0.85±1.10	1.21±1.02
Min.	‐1.99±1.00	‐2.49±0.67	‐2.02±1.02	‐0.89±1.11	‐0.79±1.22	‐0.95±1.36
Mean of |RTDs|	0.81±0.61	0.68±0.51	0.76±0.57	0.96±0.70	0.93±0.74	1.03±0.78
SD of |RTDs|	1.29±0.96	0.85±0.57	0.85±0.46	0.49±0.33	0.43±0.29	0.50±0.23

### B. Motion‐induced dose effect analysis

**Figure 2:**
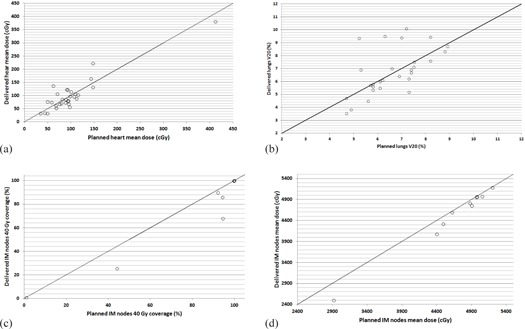
The planned and delivered dose comparison: a) heart mean dose; b) lungs V20; c) IMN 40 Gy coverage; and d) IMN mean dose.

The comparisons of the planned and delivered dose coverage for heart, lungs, and IMN are shown in Fig. 2. Each open circle represents a comparison of one patient. The descriptive statistics are summarized in Table 2

**Figure 3:**
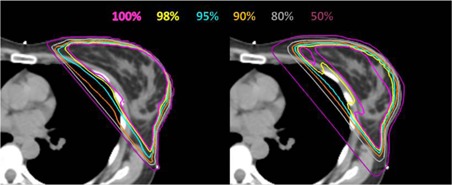
An example of the planned (left) and delivered (right) dose distribution on an axial CT image. The prescription was 267 cGy×16. The mean RTDs are (0.77, 0.36, 0.43, 0.19, −0.59, −1.00), and the SDs of the RTDs are (1.19, 0.66, 0.87, 0.55, 0.27, 0.36).

Figure 3 demonstrates an example of the planned and delivered dose distribution.

**Figure 4:**
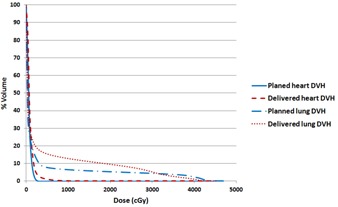
The planned and delivered heart and lungs DVH comparison for the example illustrated in Fig. 3.

Figure 4 shows the corresponding heart and lungs DVHs. The 50% line has been pushed closer to the heart and the 80% line has been pushed slightly away from the heart. The 100% and 98% isodose lines were different than the plan. Less than 1% heart and lungs DVH difference was observed in Fig. 4. We typically do not contour PTV for breast or chest wall treatment. Therefore no PTV DVH comparison can be shown.

5, 6 illustrate an example of the planned and delivered IMN 40 Gy percentage coverage (planned was 94.5%, and delivered was 68.1%) and DVHs. The planned IMN was covered entirely by the 80% isodose line. However, at least one‐third of the IMN was outside of 80% isodose line in the delivered dose distribution. The delivered IMN DVH was lower than the planned.

**Table 2:** The planned and delivered heart, lungs, and IMN dose coverages

	*Heart Mean Dose (cGy)*		*Lungs V20 (%)*		*IMN 40 Gy Coverage (%)*		*IMN Mean Dose (cGy)*	
*Planned*	*Delivered*	*Planned*	*Delivered*	*Planned*	*Delivered*	*Planned*	*Delivered*
Average	99	101	6.59	6.74	83	77	4642	4518
SD	66	66	1.19	1.79	33	36	658	781
Max.	412	381	8.92	10.08	100	100	5203	5186
Min.	35	32	4.70	3.53	1	1	2915	2503
Average Difference	2		0.15		‐6		‐123	
Average Absolute Difference	20		0.98		6		123	
Range of the Difference	[−41,76]		[−2.12,4.12]		[−26,0.1]		[−411 −11]	
SD of the Difference	28		1.45		9		140	

**Figure 5:**
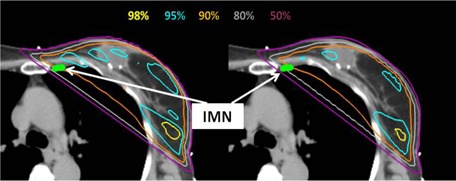
An example of the planned (left) and delivered (right) axial CT images demonstrating changes in the IMN coverage. The prescription was 200 cGy×23. The mean RTDs are (0.06, 0.15, 0.78, 0.68, −0.57, 2.76), and the SDs of the R TDs are (1.01, 1.41, 0.78, 0.52, 0.25, 0.44).

**Figure 6:**
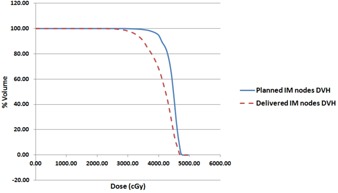
The planned and delivered IMN DVH comparison for the example illustrated in Fig. 5.

## IV. DISCUSSION

The data presented demonstrate that AlignRT system provides a reproducible setup throughout treatment, with typical breath‐hold motions of <1 mm. The dosimetric consequences of these subtle breath‐hold motions are similarly small. Gierga et al.^(15)^ performed a similar study and noted delivered and planned breath‐hold positions typically to have means around 2 mm in each direction. We used ±3 mm and ± 3° as the RTD tolerance, and the Gierga study used ±5 mm (no rotational tolerance was reported). We have simulated the motion using mean RTDs from the Gierga study, 1.0 mm SD, and their tolerance to estimate corresponding dosimetric effect. A patient (with IMN) with average motion was selected. RTDs of (2.2 mm±1.0 mm, 2.3 mm±1.0 mm, 2.0 mm±1.0 mm) and tolerance of ±5 mm were generated (0° rotational motion was used as it was not reported in the Gierga study). Delivered dose was estimated the same way in this study. In more detail, 200 entries of RTDs were simulated using the means and standard deviations listed above. Each RTD entry corresponds to an isocenter shift, and each isocenter shift results a deviated dose distribution. The final dose distribution was an equal‐weighted average of all 200 varied ones. The same orientation system shown in Fig. 1 was used for this simulation (i.e., 2.2 mm, −0.5 mm, 1 mm), which means that the isocenter moved posterior 2.2 mm, left 0.5 mm, and superior 1 mm. The estimated heart mean dose using ±5 mm tolerance was 110 cGy, the estimate using ±3 mm tolerance on the same patient was 106 cGy, and the planned dose was 97 cGy; the lungs V20 were 7.74%, 7.72%, and 6.66%; the corresponding IMN mean doses were 4983 cGy, 4970 cGy, and 4958 cGy; the corresponding IMN 40 Gy coverage were 100%, 100%, and 100%. It appears that, by increasing the tolerance, the average motion was increased as well, and it might result in slightly higher delivered heart mean dose, lungs V20, and IMN dose. A thorough study is needed to make meaningful conclusion.

Audio coaching was used for all the patients in our study. Cerviño et al.^(21)^ demonstrated an improvement in reproducibility with visual coaching vs. no coaching (0.5 mm vs. 2 mm). Vikstrom et al.^(22)^ also represented the implementation of the visual coaching. We are considering a visual feedback system in our clinic, as well.

All the DIBH patients in this study were treated in the supine position. Another way to reduce dose to the heart is to treat patients in the prone position with free breathing. The dosimetric results of these two techniques are different. Also the contralateral breast dose is often evaluated in the prone treatments. A recent study^(23)^ has shown that prone free breathing yields lower mean lung dose and V20 than supine DIBH. The mean heart dose and contralateral breast receiving >5 Gy were significantly lower in supine DIBH than prone free breathing. Although this study investigated only planned dose without considering patient motion, it still provides dosimetric information of prone free‐breathing and supine DIBH. For prone free‐breathing treatments, the interfraction motion (patient setup) is often greater than the intrafraction patient motion. Unfortunately, no study was found at this point to show the corresponding dosimetric impact.

Since both the patient setup RTDs and the treatment beam‐on/off RTDs were absolute displacements calculated against the same reference surface image throughout the treatment course, the dosimetric analysis on beam‐on portions of the RTDs provided a reasonable estimate of the breath‐hold motion effect compare to the plan. In other words, the setup uncertainties to the delivered dose do not propagate to the breath‐hold motion induced uncertainties.

There are several shortcoming of our analysis. We assumed a rigid body model (i.e., that the relative position of the chest/breast and heart was constant). This clearly is not the case. Nevertheless, this is a reasonable assumption, given the goals of the analysis. Surface has been proven to be a reasonable surrogate for DIBH patient setup from a port film analysis in Tang et al.^(20)^ A total of 270 port films from 50 patients was analyzed to assess patient surface based setup accuracy. The distance between the field edge and the anterior pericardial shadow was measured on port films and corresponding digitally reconstructed radiographs (DRR). The reported discrepancy was 0.2±0.19 cm, which was fairly reproducible. On the other hand, it was reported that the interfraction DIBH motion of the heart had reproducibility of 3 mm in the anterior–posterior (AP), 7 mm in the superior–inferior (SI), and 3 mm in the left–right (LR) directions.^(24)^ Moderate correlation was found between surface and heart setup errors in Alderliesten et al.:^(13)^ $R^{2}=0.53$, 0.37, 0.64 in AP, SI, and LR directions, respectively. Therefore, an inclusion of a more sophisticated model (i.e., deformable registration) might make the analysis more accurate. However, one should carefully evaluate the trade‐off between the complexity of the analysis and the degree of the benefit. In cases where the IMNs are targeted and where they are receiving (nearly or) full dose, they tend to be close to the field edge. If the beam is too deep, the nodal targets remain in the field, but the dose does not increase. Conversely, if the beam is too shallow, the dose can decline rapidly due to the proximity to the beam edge. Thus, the dosimetric consequences of breath‐hold motions are not symmetric (underdosage is more common than overdosage). Similarly, for structures that are well out of the planned beams, such as the heart in most of our cases, the exact reverse occurs. If the beam is too shallow, the dose remains low, and if the beam is too deep, the dose can increase (motion tends to increase the cardiac dose; which is to say that overdosage is more common than underdosage).

## V. CONCLUSIONS

Considering absolute value, the averaged mean motion during deep inspiration breath hold was smaller than or nearly 1 mm and 1°. This reflects the relative reproducibility of the patient breath hold. On average, the mean heart dose and lungs V20 are reasonably close to what has been planned. IMN 40 Gy coverage might be modestly reduced for certain cases.
